# On the predictability of postoperative complications for cancer patients: a Portuguese cohort study

**DOI:** 10.1186/s12911-021-01562-2

**Published:** 2021-06-28

**Authors:** Daniel Gonçalves, Rui Henriques, Lúcio Lara Santos, Rafael S. Costa

**Affiliations:** 1 IDMEC, Instituto Superior Técnico, University of Lisbon, Av. Rovisco Pais 1, 1049-001 Lisbon, Portugal; 2 INESC-ID, R. Alves Redol 9, 1000-029 Lisboa, Portugal; 3 LAQV-REQUIMTE, NOVA School of Science and Technology, Campus Caparica, 2829-516 Caparica, Portugal; 4 Instituto Superior Técnico, University of Lisbon, Lisbon, Portugal; 5 Experimental Pathology and Therapeutics Group of Portuguese Institute of Oncology of Porto FG, EPE (IPO-Porto), Porto, Portugal; 6 Surgical ICU of the Portuguese Institute of Oncology, Porto, Portugal; 7 Surgical Oncology Department, IPO-Porto, Porto, Portugal

**Keywords:** Machine learning, Risk prediction, Postoperative complications, Cancer, Data modeling, Clinical decision support system

## Abstract

Postoperative complications are still hard to predict despite the efforts towards the creation of clinical risk scores. The published scores contribute for the creation of specialized tools, but with limited predictive performance and reusability for implementation in the oncological context. This work aims to predict postoperative complications risk for cancer patients, offering two major contributions. First, to develop and evaluate a machine learning-based risk score, specific for the Portuguese population using a retrospective cohort of 847 cancer patients undergoing surgery between 2016 and 2018, for 4 outcomes of interest: (1) existence of postoperative complications, (2) severity level of complications, (3) number of days in the Intermediate Care Unit (ICU), and (4) postoperative mortality within 1 year. An additional cohort of 137 cancer patients from the same center was used for validation. Second, to improve the interpretability of the predictive models. In order to achieve these objectives, we propose an approach for the learning of risk predictors, offering new perspectives and insights into the clinical decision process. For postoperative complications the Receiver Operating Characteristic Curve (AUC) was 0.69, for complications’ severity AUC was 0.65, for the days in the ICU the mean absolute error was 1.07 days, and for 1-year postoperative mortality the AUC was 0.74, calculated on the development cohort. In this study, predictive models which could help to guide physicians at organizational and clinical decision making were developed. Additionally, a web-based decision support tool is further provided to this end.

**Supplementary Information:**

The online version contains supplementary material available at 10.1186/s12911-021-01562-2.

## Introduction

Cancer is a major health problem worldwide and it is among the leading death causes of the 21^st^ century. There are at least two battlefronts in reducing deaths associated to cancer, those resulting from direct consequences of the disease, and those occurring due to complications from surgery treatment [1]. Surgical complications contribute to lower survival probability and, in certain types of cancer, to aggravate the recurrence rate [1–4]. The outcome of such surgeries is still widely unpredictable due to the large number of factors involved. In an attempt to facilitate perioperative risk assessment for the selection of patients benefiting from surgery, a variety of traditional scoring systems incorporating several risk factors have been developed [5].

From a clinical perspective, the traditional risk scores (e.g., P-POSSUM [6], ARISCAT [7] and ACS score [8]) are important in choosing the course of actions, such as prehabilitation or supportive measures, to be taken during the preoperative, intraoperative and postoperative periods [5]. However, their limited predictive performance is clear, particularly in the geriatric population [9]. Moreover, most of these risk scores were constructed based on simple linear models with inherent limitations for high-dimensional and multi-variate data.

Recently, machine learning (ML) approaches for surgical outcomes prediction have been proposed. ML comprises algorithms that can learn from a set of data and improve on their own, allowing for more accurate predictions [10, 11]. For instance, Wang et al. [12] proposed several ML models to predict 5-year mortality in a bladder cancer cohort. The study used clinical and histopathological data from 117 patients, and achieved 80% accuracy. More recently, Corey et al. [13] explored ML methods to identify high-risk surgical patients from a local institution using electronic health record data. The sensitivity and specificity were 76%, evaluated across several ML models. Another example is the study conducted by Lee [14] where deep neural network models were successfully used to classify the risks of postoperative mortality, acute kidney injury, and reintubation, outperforming more traditional approaches such as Logistic Regression, ASA [15] and the Surgical Apgar [16] scores.

Despite the inherent potentialities of ongoing efforts, the existing postoperative risk prediction studies in the oncological domain are limited by the size of available hospital records, the lack of systematic evaluation of different predictive models, and no one comprehensively targets the Portuguese population. Identification of reliable prognostic factors, representative of our own patient population, may help clinicians not only to accurately select patients eligible for surgery, but also to identify high-risk patients that may benefit from individualized optimization with multimodal prehabilitation interventions. There is thus an urgent need to improve perioperative risk assessment to reduce the growing postoperative burden among patients who undergo surgery for cancer.

This work assesses the predictability of four main postoperative outcomes in cancer patients: i) existence of postoperative complications, ii) the severity of said complications, iii) the number of days in the Intermediate Care Unit (ICU), and the iv) one-year death after surgery. In this context, it offers two major contributions. First, a methodology for the prognostication of oncological postoperative outcomes. Secondly, establishes principles to support the study of this treatment, either by finding relevant variables, or improving the interpretability of these models.

## Methods

### Dataset

The data derives from a single-center retrospective cohort of cancer patients who have undertaken surgery at the Portuguese Institute of Oncology, Porto, Portugal (IPO-Porto), and were monitored from 2016 to 2018. Only surgical patients aged 18 years or older were included. All were followed up for at least one year or until death. The cohort of 847 eligible patients contains information pertaining to the demographic and physiological data, cancer location, histopathological determinants, traditional risk score variables (from P-Possum [6], ACS NSQIP [8], ARISCAT [7]), surgical procedures and outcomes of interest. From a total of 136 routinely-collected variables, only 62 are preoperative. Out of these, 20 are binary variables, 20 ordinal, 10 categorical, 5 numeric, 2 in date format and 5 are pure text variables (see Additional file 1: Table S1 in Supplementary Material). The IPO-Porto Ethics Committee approved (CES IPO:91/019) the analysis and the study of the anonymized data.

Statistical exploration of the dataset was performed in Python (version 3.8) with the aid of Seaborn^1^(version 0.11.1) and Matplotlib^2^(version 3.4.2) for the visualization, NumPy^3^(version 1.19.2) and Pandas^4^(version 1.2.1) for the data handling.

### Validation dataset

An independent validation dataset collected at IPO-Porto between January and October of 2019 was used. This cohort has the same variables as the previous dataset but only 137 patients, which have not been used for model training. There are 4 types of cancer or surgical area: head/neck, gastrointestinal, respiratory, and lymphoma, but the representativeness of the last two is residual. The average age of the patients is 61 years old; 101 patients are males and 36 are females. Additional information of the statistical analysis is provided as Supplementary Material, in Additional file 1: Figure S1.

### Data preprocessing

The preprocessing is challenged by three main issues: missing values, mixed variables with non-identical distributions and imbalanced/sparse data (considering the variety of cancers and surgery types).

*Missing values* To minimize biases and predictive uncertainty, variables with high missing rate (>40%) were removed. In less extreme cases, and whenever classifiers are unable to handle missing data, missing values were imputed using an informed method based on the k-Nearest Neighbors algorithm [17], to help reduce the error introduced when dealing with missing values.

*Categorical variable encoding* Categorical variables are commonly represented through a numeric encoding, which may not necessarily contain an implicit ordinal relationship. This quantitative or ordinal relationship might undesirably slip into the analysis. The simplest solution is to use a One-Hot encoder, consisting on splitting the categorical variable into a series of binary ones.

*Resampling* To handle the observed imbalances on some of the outcomes and avoid the bias of the classifiers towards the majority class, we apply a mixed strategy, combining synthetic oversampling with Tomek Links informed undersampling, as proposed in [18].

*Feature scaling* Numeric variables are normalized to promote the learning of the algorithms that are affected by the magnitude of the different input variables, commonly resulting in wrongfully attributed relevance.

*Feature selection* Accounting for differences on the relevance of input variables for a given outcome, a restricted number of variables were selected (according to the scheme on Addional file 1: Fig. S2, in Supplementary Material). We used the clinical expert’s opinion, to select no more than 20 preoperative variables from the dataset as inputs to the algorithms for each outcome. Filter methods offer a *p*-value representing the probability that a variable is not correlated to an outcome. We defined the *p*-value threshold at 0.0002. The $\chi ^{2}$ test is used to measure correlation for categorical variables, when the output is also categorical. The ANOVA correlation coefficient is used to measure the correlation between categorical and numeric variables (it is not relevant which one is the dependent variable). Pearson’s correlation coefficient is used when both the independent and the dependent variables are numeric.

### Outcomes

We attempt to address two main questions/outcomes: first, is a patient going to have postoperative complications? A postoperative complication was defined as a deviation from the ideal postoperative course, which is deemed clinically connected to the surgery prior, requiring any intervention, and happening within the first 90 days after the surgery for cancer treatment. Since the outcome is binary, a classification approach is used, with a discrete and well defined set of labels to attribute to a certain patient.

Secondly, how severe is the complication? The Clavien-Dindo classification system [19], in 4 major grades (excluding death), was used for the classification of surgical complications. For this outcome, a multi-class classification approach is performed.

The probability of death is also a relevant indicator to estimate the existence of future complications, and the viability of surgery for a certain patient. In this case, death might not be the result of postoperative complications exclusively, but rather a combination of factors. We conducted this outcome as a classification problem with the objective of predicting one-year mortality.

The number of days spent in the ICU following the surgical procedure represents important information for medical and hospital management reasons. Due to the continuous and purely numeric nature of this outcome, regression models are used.

### Predictive models

We implemented a set of state-of-the-art supervised ML models, and assessed the predictive performance of all.

- The classifier-based prediction algorithms were: Naive Bayes (NB), k-Nearest Neighbours (kNN), Decision Trees (DT), Random Forests (RF), Support Vector Machines (SVM), Logistic Regression (LR), Multilayer Perceptron (MLP), XGBoost Classifier (XGB) and CatBoost Classifier (CBC);
- The regression-based prediction algorithms were: Linear Regression, Ridge Regression, Lasso Regression, SVM Regressor, Elastic Regression, k-Nearest Neighbours Regressor, Decision Tree Regressor, Random Forest Regressor, XGBoost Regressor, Partial Least Squares Regression (PLS), Multilayer Perceptron Regressor and CatBoost Regressor (CBR).

All the models’ implementations were carried out using the scikit-learn [20] package (version 0.23.2) using Python (version 3.8). For the XGBoost [21] (version 1.3.3) and CatBoost [22] (version 0.24.4) algorithms two independent packages were used.

### Hyperparameter optimization

The hyperparameters of the models were selected, using informed search methods. Bayesian optimization [23] associates a probability distribution to the hyperparameters tested, making the search faster than exhaustive approaches. Two objective functions were used:

- Regression models are optimized with respect to the Root Mean Squared Error (RMSE);
- Classification models are optimized to maximize their F1-Score (the harmonic mean of precision and recall).

### Model development process

The development process was performed in two phases: training and testing using cross-validation (split into the primary dataset); independent validation (training with the primary dataset and testing on the secondary independent one, also recorded at IPO-Porto, Portugal). Both begin by preprocessing the input data before feeding it to the models, either to learn or directly predict the outputs. The difference is that in the first phase there is an intermediate step for hyperparameter optimization and in the second phase such parameters are already available.

The code and results generated in this article are available in GitHub at: https://github.com/danielmg97/cancer-prognostication-iposcore.

After model selection and optimization, a web-based graphical application for clinical context use was built using the Dash^5^ library in Python. The code repository is freely available at https://github.com/danielmg97/iposcore_webapp.

### Model performance and validation

*Classification evaluation metrics* The discrete nature of classifiers allows for simple evaluation. Given the imbalanced nature of data, accuracy is complemented with other metrics, like recall/sensitivity. The Receiver Operating Characteristic (ROC) curve can also be used to assess the model performance specifically as a measure of class separability. It is most commonly used in binary outcome settings but can be used for multi-class outcomes. In the latter, the AUC (Area Under the Curve) is more suitable and is employed in our study. The F1-score [24] combines precision and recall in a weighted average. This metric is the focus of our optimization efforts in order to guarantee the optimal sensitivity to every output class, even in multi-class settings where this measure is macro averaged. Cohen’s Kappa [25] is also used as a chance corrected standardized measure of agreement. This metric can be interpreted as follows: $\le 0$ less than chance agreement, 0.01–0.20 slight agreement, 0.21–0.40 fair agreement, 0.41–0.60 moderate agreement, 0.61–0.80 substantial agreement, 0.81–0.99 almost perfect agreement [26].

*Regression evaluation metrics* In contrast with previous confusion-based metrics, residue-based scores are used to assess the predictability of numeric outcomes. RMSE is a quadratic scoring rule that also measures the average magnitude of the error. Since the errors are squared before they are averaged RMSE gives a larger weight to larger errors. The mean absolute error (MAE) measures the average magnitude of the errors on a set of predictions, complementing RMSE. Apart from checking the absolute fitment of the model, the Coefficient of Determination, or $R^{2}$, is also used to assesses the fitness of the model to the available (training) data.

*Model validation* We applied ten-fold cross-validation (10 mutually exclusive test sets, each composed by 10% of the total patients) to assess the models’ ability to generalize into unseen data and also its performance variability, by testing in various sets of instances.

*External validation* The models were validated on an independent cohort with 137 patients’ registries from the same hospital.

## Results

In this study, we tested the predictive performance of ML models for four main postoperative outcomes derived from our cancer patient population, in order to to facilitate prehabilitation strategies and manage hospital resources more efficiently.

### Data exploration

**Fig. 1:**
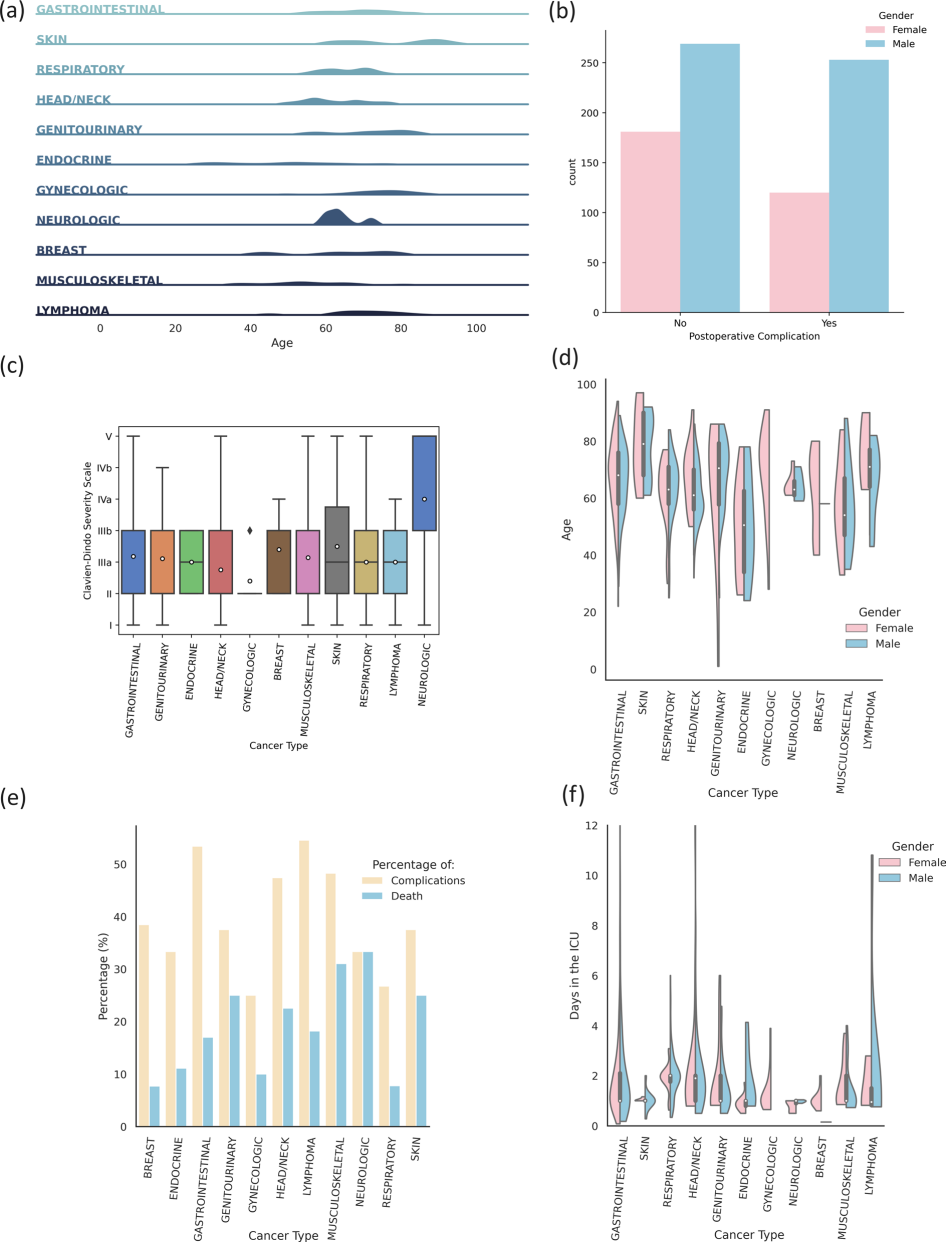
Cohort data overview: **a** cancer type density plot according to patient age **b** postoperative complications by gender **c** complications’ severity by cancer type **d** age distribution by gender and cancer type **e** percentage of complications/deaths by cancer type **f** distribution of days in the ICU by cancer type and gender

Figure 1 displays the summary of the cohort data. The available cohort is constituted by four major surgical types: thoracic (13.91%), digestive (40.87%), head and neck (22.98%), and others (22.24%). Of all surgeries, 43.83% are related to gastrointestinal cancers, 21.21% head/neck, 14.02% respiratory, 5.69% genitourinary, 3.32% muscoloskeletal, 2.36% gynecologic, 2.23% endocrine, 1.99% skin, 1.61% breast, 1.36% neurologic, and 1.24% were lymphomas. The surgeries’ type was mainly elective and only 11% of the procedures correspond to emergency surgeries.

The majority of patients in this cohort (49.57%) have primary malignant tumours and less than 1% have benign tumours. Considering metastization, 27.7% of the patients have malignant tumours with nodal metastasis, and 20.87% have malignant tumours with distant metastasis.

The therapeutic profiling of these patients can be detailed by analyzing neoadjuvant therapy options, such as chemotherapy. In our population, 27% of the patients have been subjected to this kind of treatment.

There are 11 types of cancer present and the incidence is mainly concentrated on older people, closer to the age of 65 (Fig. 1a). There are more men undergoing surgery and they are also more likely to develop postoperative complications than women (as shown in Fig. 1b). There are types of cancer more likely to complicate and more lethal than others (Fig. 1e), where Neurologic and Musculoskeletal cancers are portrayed as the most lethal types. The degree of Clavien-Dindo severity [19] associated to the postoperative complications is similar across the different types (shown in Fig. 1c), where Neurologic cancers are portrayed as the type with more severe complications. The days in the ICU rarely exceed 2 to 3 days but can stretch as far as 2 weeks or more (Fig. 1f).

### Postoperative complications

Table 1 shows the performance of the top 5 models for the postoperative complication outcome. It can be observed that it is possible to predict the presence of postoperative complications with 65% accuracy and 0.69 AUC by a Random Forest (RF) using 8 input variables (Addional file 1: Table S1—Supplementary Material) after the feature selection process: ASA score, ACS functional status, ACS systemic sepsis, ACS dyspnea, PP respiratory, PP hemoglobin, PP number of procedures, and PP peritoneal contamination. Other models are able to achieve similar predictive performance, but are outperformed by the RF that can be a more easily interpretable solution upon individual tree analysis, when compared with alternatives such as the MLP model.

Furthermore, as proposed in our methodology, the models were validated in an independent set of 137 patients. The RF achieved an accuracy of 67% and an AUC value of 0.71 , and the overall metrics achieve higher results, supporting the generalization ability of our solution.

**Table 1:** Top 5 models for the postoperative complications outcome, obtained through cross-validation inside the primary 847 patients dataset

Model	Kappa	Recall	AUC	F1-Score	Accuracy
RF	0.293 ± 0.095	0.645 ± 0.081	0.691 ± 0.057	0.645 ± 0.046	0.652 ± 0.048
MLP	0.285 ± 0.096	0.642 ± 0.101	0.663 ± 0.053	0.641 ± 0.050	0.648 ± 0.048
SVM	0.282 ± 0.121	0.640 ± 0.098	0.676 ± 0.058	0.640 ± 0.060	0.646 ± 0.061
CBC	0.276 ± 0.109	0.636 ± 0.131	0.681 ± 0.064	0.635 ± 0.055	0.646 ± 0.053
LR	0.272 ± 0.086	0.634 ± 0.140	0.685 ± 0.056	0.632 ± 0.041	0.645 ± 0.044

The values are the mean ± standard deviation (SD)

### Severity of complications

The complications’ severity was the second outcome of interest. Table 2 compares the predictive performance of the top 5 models. Overall, the predictability is in line with expectations for a 4 degree scale in a very imbalanced setting, with underrepresented grades. Being a harder prediction task, the feature selection process considered a higher amount of variables when compared with other outcomes, using 15 of the total 20 inputs (Addional file 1: Table S1—Supplementary Material): ASA score, ACS functional status, ACS systemic sepsis, ACS dyspnea, ARISCAT preoperative anemia, ARISCAT emerging procedure, PP respiratory, PP ECG, PP arterial pulse, PP hemoglobin, PP leukocytes, PP urea, PP sodium, PP number of procedures, and PP peritoneal contamination. Of all the models tested, RF had higher predictive ability with an accuracy of 51% and 0.65 AUC, when compared to other models.

In the independent validation set, the RF model was able to predict the outcome with similar results (accuracy = 61% and AUC = 0.84).

**Table 2:** Top 5 models for the complication’s severity outcome, obtained through cross-validation inside the primary 847 patients dataset (mean ± SD)

Model	Kappa	Recall	AUC	F1-Score	Accuracy
RF	0.225 ± 0.127	0.431 ± 0.164	0.651 ± 0.083	0.410 ± 0.093	0.506 ± 0.081
CBC	0.197 ± 0.098	0.430 ± 0.239	0.634 ± 0.089	0.377 ± 0.082	0.434 ± 0.071
DT	0.185 ± 0.118	0.388 ± 0.254	0.620 ± 0.094	0.368 ± 0.095	0.465 ± 0.083
SVM	0.157 ± 0.096	0.431 ± 0.243	0.642 ± 0.069	0.357 ± 0.062	0.393 ± 0.055
XGB	0.158 ± 0.128	0.424 ± 0.221	0.629 ± 0.062	0.354 ± 0.103	0.379 ± 0.091

### Days in ICU

The prediction of days spent in the ICU is a difficult task given the typical short stays of 1 or 2 days, contrasting with a small percentage of patients with longer stays. Although various transformations were used to attempt to minimize the effects of the imbalance in the data, the regressors predict lower values. Ridge regression showed superior performance (MAE of approximately 1 day) compared with the other models (Table 3).

After the independent validation, the results of the best model remained identical (MAE of 1.07, RMSE of 1.77 and R^2^ of 0.07).

The feature selection process indicated 7 relevant input variables (Addional file 1: Table S1—Supplementary Material), which might mean a reduced data extraction effort for the clinicians in the future: ACS systemic sepsis, ACS acute renal failure, ARISCAT respiratory infection, ARISCAT preoperative anemia, ARISCAT surgery duration, ARISCAT emerging procedure, PP number of procedures.

**Table 3:** Top 5 models for the days in the ICU outcome, obtained through cross-validation inside the primary 847 patients dataset (mean ± SD)

Model	MAE	RMSE	R^2^
Ridge	1.071 ± 0.161	1.724 ± 0.436	0.042 ± 0.105
Linear	1.080 ± 0.157	1.729 ± 0.424	0.030 ± 0.122
PLS	1.079 ± 0.153	1.730 ± 0.420	0.029 ± 0.116
MLPR	1.075 ± 0.157	1.732 ± 0.426	0.029 ± 0.104
RF	1.077 ± 0.151	1.735 ± 0.428	0.027 ± 0.099

### One-year mortality

The results of the ML models for one-year mortality prediction are presented in Table 4. Overall, for the best mortality risk classifier the accuracy of prediction is 85% and the AUC value is 0.74, which outperforms other tested models.

The data exploration process revealed the severe imbalance of 1:8, towards the negative result for 1 year death. However, this imbalance was not critical since there were still close to 100 patients representing the minority class and resampling techniques were viable in this binary classification setting. This outcome only makes use of 7 input variables (Addional file 1: Table S1): ASA score, ACS functional status, ACS systemic sepsis, ACS weight, PP hemoglobin, PP peritoneal contamination, PP state of malignancy.

The accuracy in the validation cohort was similar to that of the development cohort with an accuracy of 85% and an AUC of 0.74.

**Table 4:** Top 5 models for the one-year death prediction outcome, obtained through cross-validation inside the primary 847 patients dataset (mean ± SD)

Model	Kappa	Recall	AUC	F1-Score	Accuracy
RF	0.371 ± 0.09	0.649 ± 0.292	0.735 ± 0.07	0.683 ± 0.046	0.845 ± 0.026
CBC	0.364 ± 0.13	0.669 ± 0.265	0.727 ± 0.073	0.681 ± 0.066	0.837 ± 0.036
XGB	0.345 ± 0.088	0.652 ± 0.283	0.718 ± 0.059	0.67 ± 0.044	0.838 ± 0.032
SVM	0.313 ± 0.091	0.664 ± 0.221	0.746 ± 0.059	0.656 ± 0.046	0.803 ± 0.028
NB	0.296 ± 0.094	0.671 ± 0.165	0.744 ± 0.041	0.644 ± 0.049	0.772 ± 0.044

### Knowledge extraction via associative models

Given the competitive results of associative models, together with their unique knowledge extraction capabilities, further studies were conducted on these models. As an extension to the results obtained from this study, an improvement over traditional model representation is proposed.

**Fig. 2:**
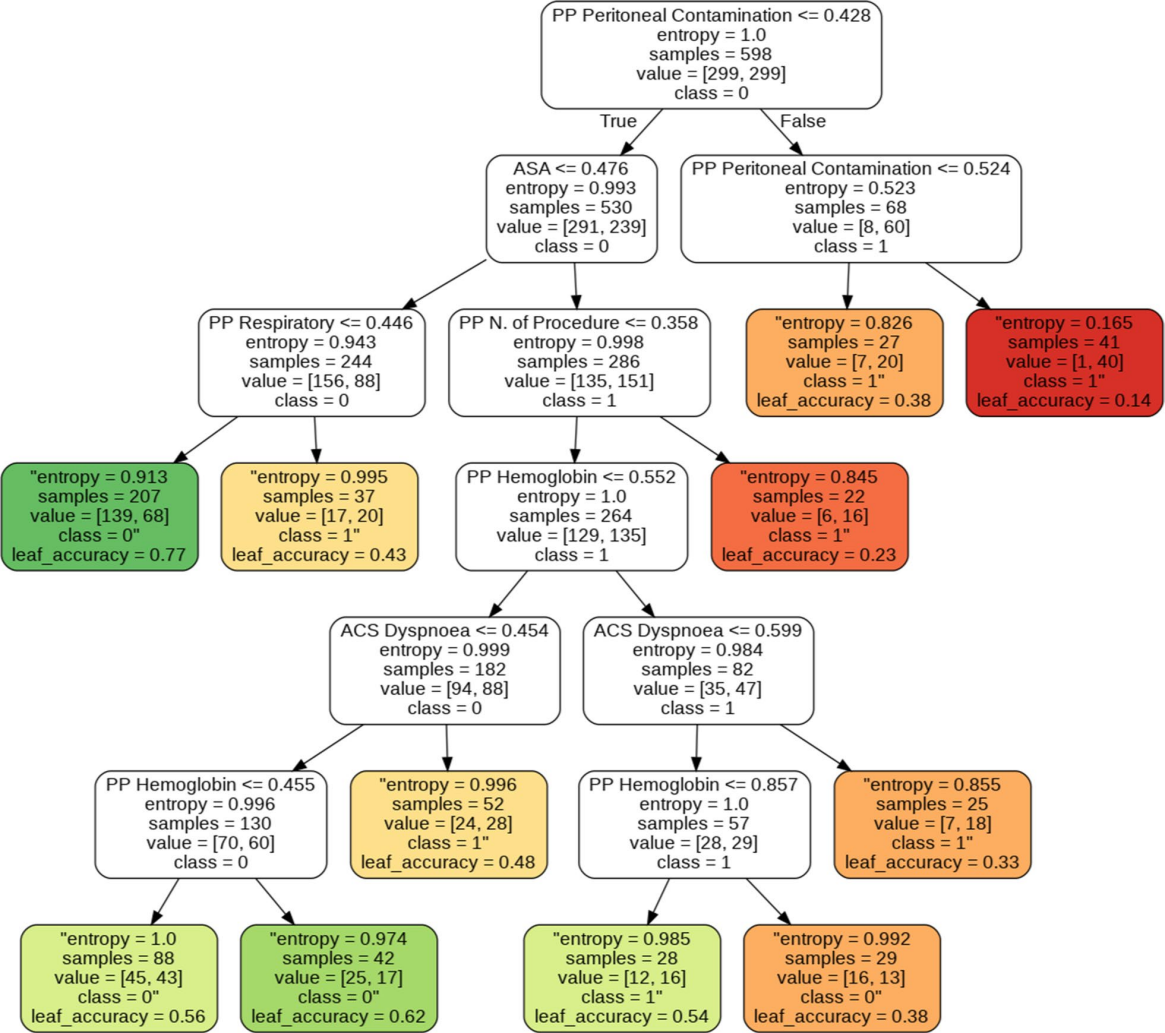
Example of a Decision Tree for the “postoperative complication” outcome. The uncolored boxes indicate decision nodes. The colored boxes represent the the leaves, meaning the output (greener denotes smaller error)

The test set error is calculated for each node individually and displayed at leaf level. Additionally, leaf nodes are colored, traducing the error degree associated to the validation process (Fig. 2).

This specific type of visualization and can be further extended, allowing for a quick assessment of the decision process and improving interpretability. This representation further helps doctors in the knowledge extraction process and in assessing the confidence level on the association rules captured by the models, and will eventually be implemented in tools used at the hospitals. An illustrative example is presented in Fig. 2, based on a Decision Tree used to predict the existence of complications. The full results for all outcomes are given as supplementary material available in the GitHub repository.

### Variables importance

**Fig. 3:**
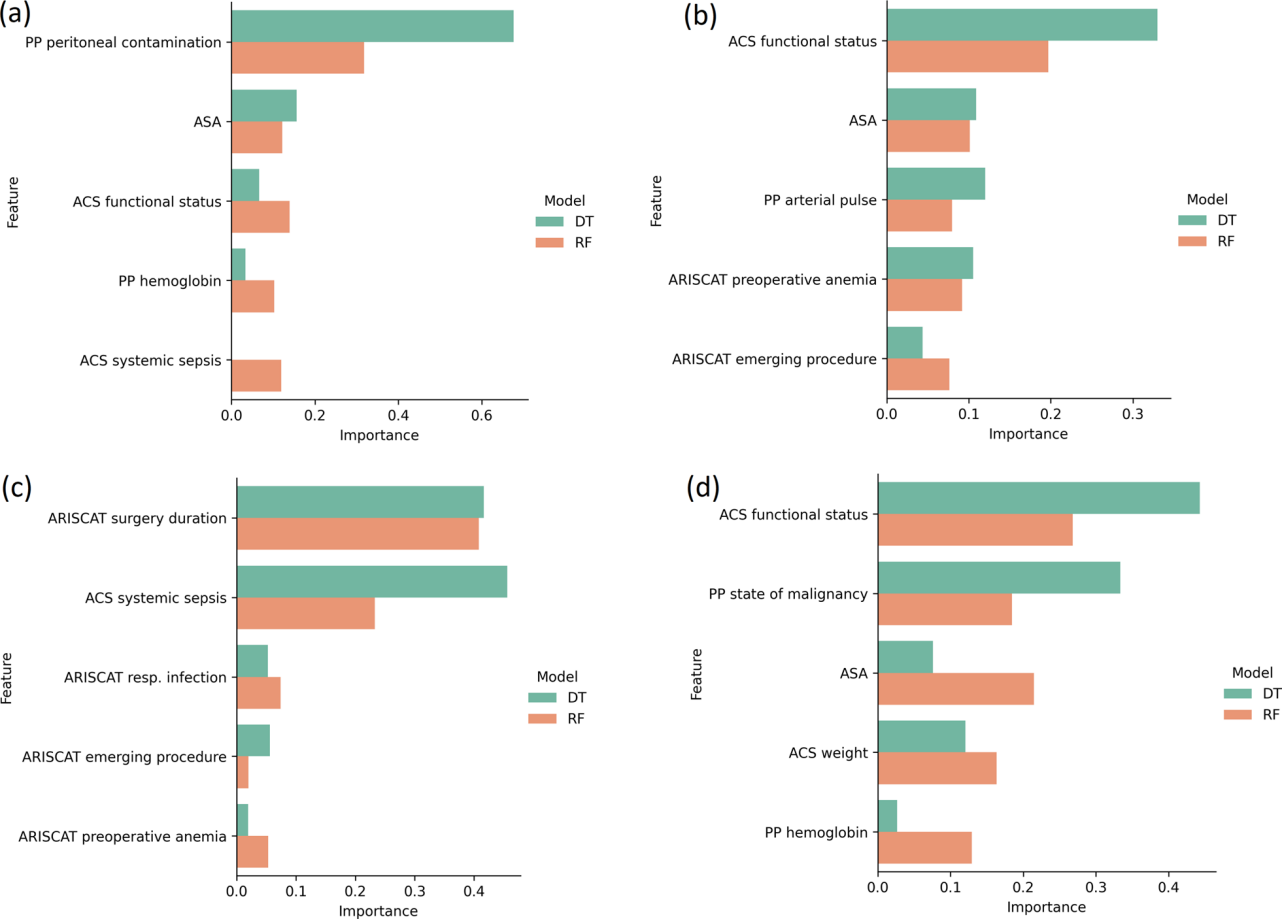
The top 5 variables with the highest feature importance (according to Decision Trees and Random Forests) for: **a** existence of postoperative complications prediction **b** severity of complications prediction **c** days in the ICU prediction **d** 1-year death prediction

Tree-based models not only stand out for their intuitive representation, but also for offering information about the importance of each feature in the prediction process. This information might be relevant for physicians in order to reduce the variable collection effort. Currently, IPO-Porto is collecting more than 60 pre-operative variables, but not all seem to be of importance for the predictions. These models can indicate the relative feature importance for each input variable when making a prediction. A tool that is understandable and transparent contributes to an easier adoption and improved clinical decision confidence. Figure 3 shows the feature importance information for the Decision Trees (DT) and Random Forest (RF) models.

For these models, the importance of a feature in the decision process directly traduces the utility of a variable when branching a node. For instance, peritoneal contamination is seen as relevant indicator for the prediction of postoperative complications (Fig. 3a). It is the first decision that will be made at the root of the Decision Tree and the split generated by this node will lead to 2 major groups of patients. One with more probability of complications than the other. Within these subgroups, there are other decisions to make, continuing the splitting process and increasing the detail level to a point where the model is more certain about the most probable outcome for a certain patient.

### Clinical decision support system

**Fig. 4:**
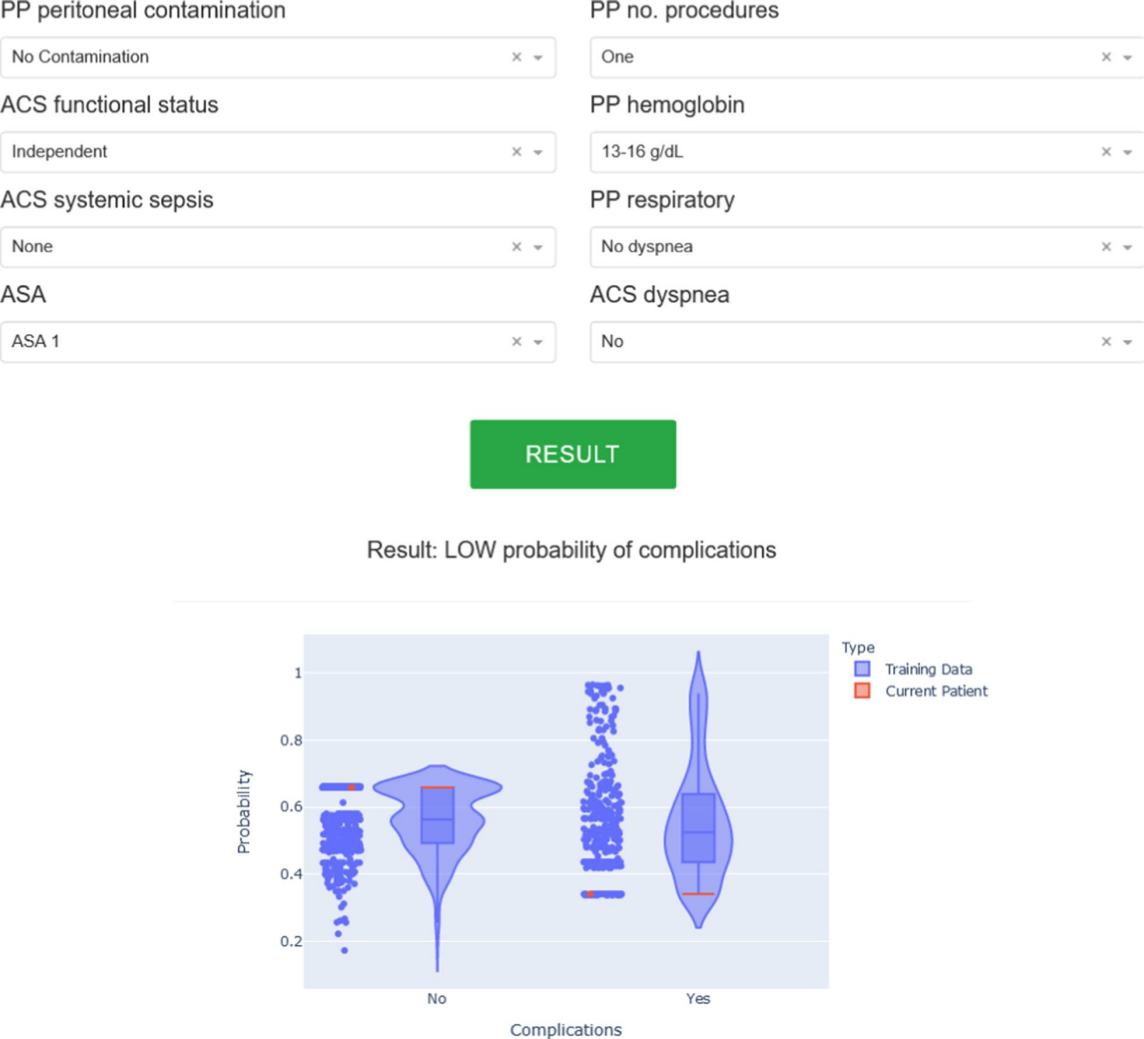
Screenshot example of the ’Existence of Complications’ tab from the web application. This outcome requires 8 input variables for a patient and predicts a probability

Finally, we developed a web-based tool to facilitate the usability of the selected models. The serialized predictive models can be used by clinicians in order to assess cancer patients in preoperative context, after adding the variables required for each outcome. The user can then easily obtain the output of the models via a graphical interface using the ‘Result’ button. For the classification tasks, the predicted probabilities for the training set are plotted, as well as the probability for the current patient, to enable comparisons and further understand the confidence of the model. The output is chosen based on the probabilities dealt by the predictive model, by choosing the outcome with the highest probability among the range of possibilities. For the regression tasks, two graphs are plotted. One with the actual values versus the predicted values of the model, and a plot of the predictions’ residuals, both using the training data (see example in Fig. 4). The web application is freely available at https://iposcore.herokuapp.com/.

## Discussion

The importance of surgical risk stratication to guide interventions is well known. In this study, we investigated the use of machine learning techniques in the surgical risk prediction of cancer patients.

Although the clinical application of ML to the postoperative complications domain has been relatively limited, in the last years, an increasing number of works have been proposed. For example, Bihorac et al. [27] predicted postoperative complications, with AUC values ranging from 0.82 to 0.94, in a cohort of 51,457 patients. Corey et al. [13] also employed ML methods to predict a similar outcome, using a cohort of 66,370 patients, obtaining AUC values ranging from 0.75 and 0.92, a sensitivity of 0.78 and a specificity of 0.75. This is similar to our best risk models, and will potentially be helpful to complement medical prognosis for cancer patients undergoing surgery in the Portuguese hospitals.

Furthermore, IPO-Porto previously developed a simple Logistic Regression model, *MyIPOrisk-score* [28], based on the Age, Gender, P-Possum (Physiological) score and ACS NSQIP (serious complications) score to predict the probability of developing postoperative complications. This study was developed using 341 digestive cancer patients and obtained an AUC value of 0.808 for the same set of patients. While we could not calculate the AUC (due to only having the binary output available), this traditional score performed inferiorly to the RF model for the 137 independent evaluation patients (accuracy = 0.613, F1-score = 0.101 and Cohen’s Kappa = 0.044).

For the complications’ severity prediction, Burke et al. [29] targeted only grades IV and V (life-threatening and requiring intensive care unit management or death) of complications’ severity for 30 days after non-elective cholecystectomy. This study uses Logistic Regression to predict the risk level (low, medium or high) of surgical complications resulting in Clavien-Dindo IV and V grades. The results point to an AUC value of 0.87 in the validation set. These results can not be directly compared, but can be considered to be in line with our study.

Predicting the days in the Intermediate Care Unit (ICU), can be an important part of predicting the length of hospital stays, allowing for better resource allocation. The studies found are generally aimed at predicting the total hospital stay length (including the various units where a patient might be) or at predicting the stays in Intensive Care Units. The number of days in the ICU is typically short, but these stays can stretch as far as 2 weeks. Our best models are able to predict this duration with an error close to 24 hours which could constitute critical information either for clinical or management reasons, allowing for better resource allocation and to manage patient’s and doctor’s expectations.

For the mortality prediction, previous studies have attempted to predict similar outcomes. Wang et al. [12] predicted 5-year mortality in a bladder cancer cohort of 117 patients with 0.8 accuracy, 0.86 sensitivity and 0.72 precision. Similarly, Corey et al. [13] included the prediction of 30-day mortality, with an AUC of 0.92, using information from 66,370 patients. Furthermore, Bihorac [27] predicted mortality for 1, 3, 6, 12 and 24 months after surgery with an AUC ranging from 0.83 for 1 month, to 0.77 for 24 months mortality. Although it is impossible to establish direct comparisons, due to cohorts and study characteristics, our model offers competitive and potentially relevant results for the the Portuguese population.

**Fig. 5:**
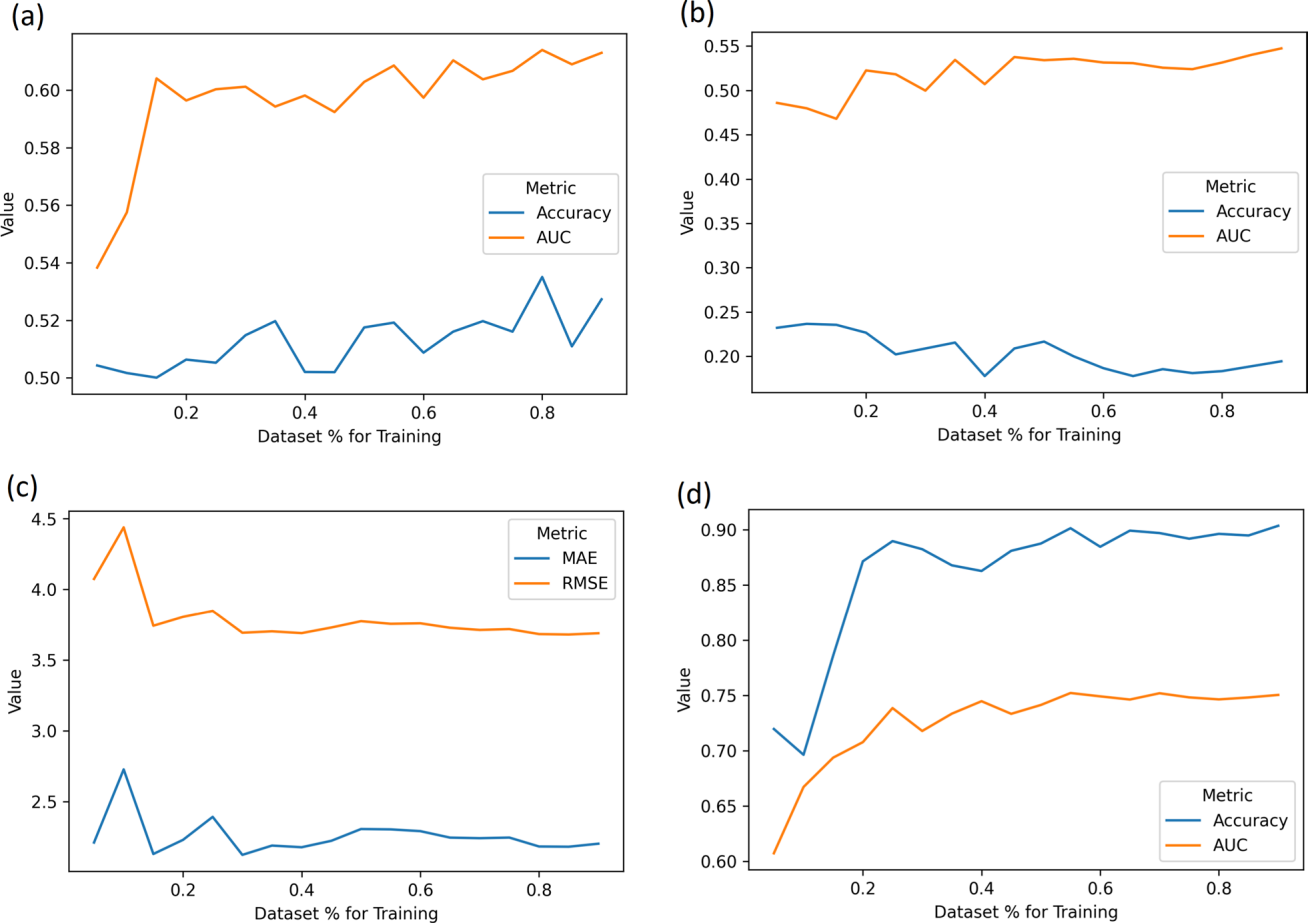
Performance evolution according to training set size: **a** postoperative complications prediction **b** complications’ severity prediction **c** days in the ICU prediction **d** 1-year death prediction

Finally, the design of this study is conditioned by the quality of the data. The available dataset is considerably smaller when compared with studies such as Corey et al. [13]. To study the impact of the training set size, a simple Naive Bayes (for complications, severity and death prediction) and a Linear Regression (for the days in the ICU) were used to assess predictive performance according to the number of patients used in training. The original dataset was used for training allowing only a predetermined percentage to be fed to the models. The 137 patients in the independent dataset were used to maintain a stable testing process. Figure 5 shows how each model performed in the fixed test set, when trained with increasingly larger portions of the main dataset of 847 patients. Even when considering the prediction of the complications’ severity - a harder task due to the cardinality of the outcome - the performance (collected under a cross-validation scheme) stabilizes after observing 50% of the population. This seems to indicate that the available dataset is sufficient.

## Conclusions

In this work, we applied machine learning models for assessing the predictability of four major cancer surgical outcomes, with the goal of increasing the accuracy of previous traditional risk scores. We demonstrated that machine learning models derived from our single-center cohort were able to improve the accuracy of a previous traditional risk score. For these predictive models, we developed a web-based clinical decision support application based on few variables as input, that can be used by physicians. Model interpretability is also enhanced, by offering new visualization options for tree-based models, in order to support medical decision processes. Additionally, information about relevant variables for the outcomes prediction is provided, contributing to more efficient data acquisition processes.

The main limitations of the present work are: i) missing values in the dataset, requiring imputation, ii) possible difficulties on algorithms training due to the limited single-center cohort size, iii) the independent validation was performed in a local set of patients only and iv) the web tool was not tested with multi-center data.

With the ongoing monitoring of new patients, the cohort study will increase in size, which can contribute to improve the predictability of imbalanced outcomes.

## Supplementary Information

**Additional file 1**. Supplementary materials regarding the training and validation datasets characterization.

## Data Availability

The dataset used during the current study is not publicly available due to data privacy concerns but is available from the corresponding author on reasonable request.

## Footnotes

1 https://seaborn.pydata.org/.

2 https://matplotlib.org/.

3 https://numpy.org/.

4 https://pandas.pydata.org/.

5 https://plotly.com/.
