# Supplementary material for: On the predictability of postoperative complications for cancer patients: a Portuguese cohort study
Source: BMC Med Inform Decis Mak. 2021 Jun 28;21:200. doi: 10.1186/s12911-021-01562-2 (PMC8237481; doi:10.1186/s12911-021-01562-2)
Supplement: Supplementary file 1 — Additional file 1. Supplementary materials regarding the training and validation datasets characterization. [file 12911_2021_1562_MOESM1_ESM.pdf]

Supplementary Material
Study Repository (Code and Results)
https://github.com/danielmg97/cancer-prognostication-iposcore

Web Tool
Link: https://iposcore.herokuapp.com/
Repository: https://github.com/danielmg97/iposcore_webapp

Description of Data:

Table S1: Summary of cohort data characteristics: input variables and output variables (star-marked).
† Nominal values assumed to have an explicit ordering for value disclosure.

Domain	Variable	Type	min†	Max†	Target
PATIENT DATA	age	numerical	22	97	{ }
	genre	binary	female	male	{ }
	pre-operatory comorbidities	text	–	–	{ }
ADMISSION REQUEST	anesthesia request date	date	–	–	{ }
INTERNMENT	surgery type	binary	elective surgery	urgent	{ }
	specialty	text	thoracic	other	{ }
	days at the ICU*	numerical	0.1	18.0	{ }
	surgery recurrence	binary	no	yes	{ }
	preoperative QT	binary	no	yes	{ }
SURGERY DATA	ASA	ordinal	healthy patient	dying patient	{1,2,4}
	location	categorical	abdomen	vulva	{ }
	preoperative diagnosis	text	–	–	{ }
	surgery date	date	–	–	{ }
	specialty COD	categorical	digestive	thoracic –	{ }
	procedures: COD	text	–	–	{ }
ACS RISK	ACS age	categorical	<65	>85	{ }
	ACS procedure	text	–	–	{ }
	ACS functional status	ordinal	independent	total dependency	{1,2,4}
	ACS emergency	binary	no	yes	{ }
	ACS diabetes	ordinal	no	insulin	{ }
	ACS hypertension	binary	no	yes	{ }
	ACS ICC	binary	no	yes	{ }
	ACS smoker	binary	no	yes	{ }
	ACS ASA	categorical	healthy patient	dying patient	{ }
	ACS steroids	binary	no	yes	{ }
	ACS ascites	binary	no	yes	{ }
	ACS systemic sepsis	ordinal	none	septic shock	{1,2,3,4}
	ACS ventilation dependency	binary	no	yes	{ }
	ACS disseminated cancer	binary	no	yes	{ }
	ACS dyspnea	ordinal	no	when resting	{1,2}
	ACS DPOC	binary	no	yes	{ }
	ACS dialysis	binary	no	yes	{ }
	ACS acute renal failure	binary	no	yes	{3}
	ACS height	numerical	137	193	{ }
	ACS weight	numerical	36	169	{4}
ARISCAT	ARISCAT age	ordinal	<51	>80	{ }
	ARISCAT SpO2	numerical	>95%	<91%	{ }
	ARISCAT resp. infection	binary	no	yes	{3}
	ARISCAT preoperative anemia	binary	no	yes	{2,3}
	ARISCAT surgical incision	categorical	peripheral	intrathoracic	{ }
	ARISCAT surgery duration	ordinal	<2	>3	{3}
	ARISCAT emerging procedure	binary	no	yes	{2,3}
P-POSSUM	PP age	categorical	<61	>70	{ }
	PP respiratory	ordinal	without dyspnea	resting dyspnea/fibrosis	{1,2}
	PP ECG	ordinal	normal ECG	other abnormal rhythms	{2}
	PP systolic blood pressure	ordinal	110-130mmHg	<90mmHg	{ }
	PP arterial pulse	ordinal	50-80 bpm	<40 or >120 bpm	{2}
	PP hemoglobin	ordinal	13-16 g/dl	<10 or >18 g/dl	{1,2,4}
	PP leukocytes	ordinal	4-10	>20 or <3	{2}
	PP urea	ordinal	<7.6	>15	{2}
	PP sodium	ordinal	>135 mmol/l	<126 mmol/l	{2}
	PP potassium	ordinal	3.5-5 mmol/l	<2.9 or >5.9 mmol/l	{ }
	PP glasglow scale	categorical	15	<9	{ }
	PP type of surgery	categorical	minor surgery	major/complex surgery	{ }
	PP number of procedures	ordinal	1	>3	{1,2,3}
	PP CEPOD-classification	categorical	elective	emergency surgery (<2h)	{ }
	PP peritoneal contamination	ordinal	no contamination	intestine/pus/blood	{1,2,4}
	PP state of malignancy	ordinal	not malignant	malignant with metastasis	{4}
	PP cardiac	ordinal	no heart failure	jugular venous pressure	{ }
POST SURGICAL COMPLICATION	post-surgical complication*	binary	no	yes	{ }
	Clavien-Dindo classification*	categorical	0	7	{ }
DISCHARGE	death up to 1 year*	binary	no	yes	{ }

Target **1** = Existence of Complications; Target **2** = Severity of Complications; Target **3** = Days in the ICU; Target **4** = Death After 1 Year.

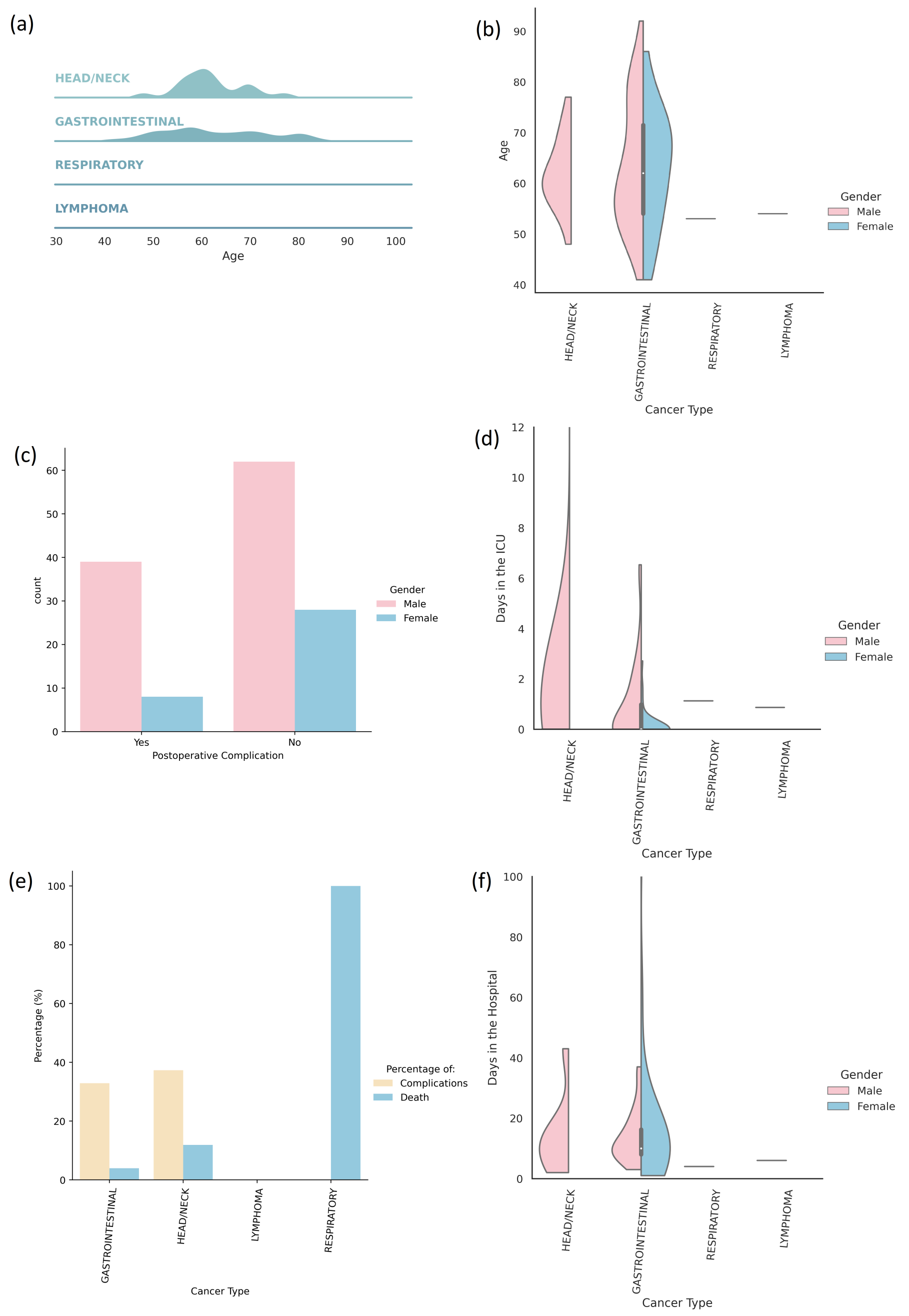

Figure S1: **Validation dataset overview: (a)** Cancer type density plot according to patient age **(b)** Age distribution by gender and cancer type **(c)** Complications' count by gender **(d)** Distribution of days in the ICU by cancer type and gender **(e)** Percentage of complications/deaths by cancer type **(f)** Distribution of total hospital stay by cancer type and gender.

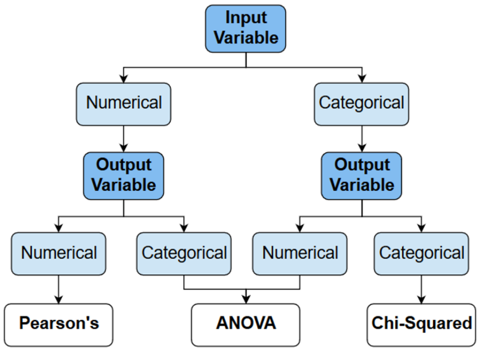

Figure S2: Scheme of the feature selection techniques used depending on the type of variables.
